# RAS gene polymorphisms, classical risk factors and the advent of coronary artery disease in the Portuguese population

**DOI:** 10.1186/1471-2261-8-15

**Published:** 2008-07-17

**Authors:** Ana I Freitas, Isabel Mendonça, Maria Brión, Miguel M Sequeira, Roberto P Reis, Angel Carracedo, António Brehm

**Affiliations:** 1 Human Genetics Laboratory, University of Madeira, Portugal; 2 Research Unit, Central Hospital of Funchal, Portugal; 3 Centro Nacional de Genotipado (CEGEN), Instituto de Medicina Legal, Universidad de Santiago de Compostela, Spain; 4 Department of Biology, University of Madeira, Portugal; 5 Medical Sciences Faculty, Universidade Nova de Lisboa and Pulido Valente Hospital, Lisbon, Portugal

## Abstract

**Background:**

Several polymorphisms within the renin-angiotensin system cluster of genes have been associated with the advent of coronary artery disease (CAD) or related pathologies. We investigated the distribution of 5 of these polymorphisms in order to find any association with CAD development and distinguish if any of the biochemical and behavioural factors interact with genetic polymorphisms in the advent of the disease.

**Methods:**

*ACE *I/D (rs4340), *ACE *A11860G (rs4343), *AT1R *A1166C (rs5186), *AGT *T174M (rs4762) and *AGT *M235T (rs699) gene polymorphisms were PCR-RFLP analysed in 298 CAD patients and 510 controls from Portugal. Several biochemical and behavioural markers were obtained.

**Results:**

*ACE *I/D DD and *ACE*11860 GG genotypes are risk factors for CAD in this population. The simultaneous presence of *ACE *I/D I and *ACE*11860 A alleles corresponds to a significant trend towards a decrease in CAD incidence. We found several synergistic effects between the studied polymorphisms and classical risk factors such as hypertension, obesity, diabetes and dyslipidaemia: the presence of the DD genotype of *ACE *I/D (and also *ACE*11860 GG) increases the odds of developing CAD when associated to each one of these classical risk factors, particularly when considering the male and early onset CAD subgroup analysis; *AGT*235 TT also increases the CAD risk in the presence of hypertension and dyslipidaemia, and *AT1R*1166 interacts positively with hypertension, smoking and obesity.

**Conclusion:**

*ACE *polymorphisms were shown to play a major role in individual susceptibility to develop CAD. There is also a clear interaction between RAS predisposing genes and some biochemical/environmental risk factors in CAD onset, demonstrating a significant enhancement of classical markers particularly by *ACE *I/D and *ACE*11860.

## Background

Cardiovascular diseases represent today the main cause of death in human adults in western populations. A reasonable number of studies have focused in testing specific genetic markers among groups of ischemic coronary artery disease (CAD) patients and control groups, aiming to find a correlation between these gene polymorphisms and disease. The renin-angiotensin system (RAS) has been shown to play a key role in the regulation of blood pressure and influence the cardiovascular system [1], and several genes belonging to this system have been associated with CAD. Two of the most intensively investigated genetic polymorphisms are the insertion/deletion (I/D) alleles of the angiotensin I-converting enzyme (*ACE*) gene and mutations at the angiotensin II AT1 receptor. Several mutations at the angiotensinogen (*AGT*) gene have also been studied as candidates in essential hypertension [2,3] or myocardial infarction (MI) [4].

Albeit all these and other markers that have been identified in latter years as possible risk factors for several pathologies associated to CAD, a considerable number of opposing results also exist. The DD genotype of *ACE *for instance has been found to be linked to MI [5,6] but contrary reports also exist [7,8]. There are even conflicting reports on the assessment of risk profiles depending on the populations studied [6,9]. The *AT1R *A1166C polymorphism has also been subjected to opposite reports as to its role in CAD, particularly when it is related to the patient's geographic origin: it was found to be significantly associated with CAD in Caucasians [9,10] but not in Asians [11]. The *AGT *M235T polymorphism has been shown to be positively related to essential hypertension [12] and MI [9] but other studies found no relation at all between the marker and disease [13,14] and few reports exist to confirm an association with plasmatic AGT concentration level [14].

In this report we have studied the distribution of genotypic and allelic frequencies at 5 loci, in two groups of individuals from Madeira Island (Portugal), an island settled mainly by the Portuguese. A group of patients with a known CAD history and a control group with no CAD related pathologies were selected. The main focus was to analyse these genetic polymorphisms, alone or combined in haplotypes, and clarify their potential association with CAD related conditions. Knowing that in most cases CAD has a multifactorial basis, involving a number of genes and environmental factors interacting to determine whether or not the disease will develop, we also tried to determine a possible interaction between the five gene polymorphisms and several well known CAD linked factors.

## Methods

The total population of this study consisted of 808 Caucasian individuals (aged between 18 and 70 years old) divided in two groups: 510 subjects without a history of CAD, MI, or stroke – excluded after a medical examination and interview – randomly selected from the electoral rows, who participated as controls, plus 298 individuals recruited from patients admitted to the Cardiology Care Unit of the Main Hospital of Funchal (Hospital Central do Funchal, Madeira Island, Portugal). Patients' recruitment satisfied the following criteria: stable coronary disease suggested by clinical analysis and proved by angiographic exam (a significant lesion was considered when ≥ 75% of luminal narrowing was observed in at least one of the major arteries) or occurrence of MI as defined by the WHO criteria. This investigation is in conformity with the principles outlined in the Declaration of Helsinki and was approved by the Hospital's Ethics Committee; all subjects gave their informed consent. Cases and controls filled in a questionnaire about their personal histories – age, sex, essential hypertension, diabetes mellitus, smoking habits, overweight, sedentary habits, alcohol ingestion and family medical history – and provided blood samples for genotype analysis and biochemical measurements. The presence of traditional risk factors in both groups was determined using the criteria standardized by the European Society of Cardiology: an hypertensive condition was attributed when systolic blood pressure values were ≥ 139 mm Hg and/or diastolic blood pressure values ≥ 89 mm Hg in at least two separate measurements or when being medicated against hypertension; subjects were considered smokers when consuming more than five cigarettes per day or non-smokers when never smoked or had stopped smoking at least one year before sample collection; obesity was defined for BMI values ≥ 30 kg/m^2^; dyslipidaemia was considered for plasmatic values of total cholesterol ≥ 200 mg/dl, triglycerides ≥ 150 mg/dl, LDL ≥ 130 mg/dl and HDL ≤ 40 mg/dl. Table 1 gives the basic characteristics of the studied population.

**Table 1:** Baseline characteristics and clinical data for conventional risk factors

	Control	CAD	P
n	510	298	
Gender (Male/Female, %)	57.84/42.16	78.86/31.82	***
Age (years, mean)	47.47 ± 12.55	54.96 ± 10.40	***
Familiar CAD history (%)	12,16	56,04	***
Sedentarism (%)	61.96	58.39	NS
Smoking habit (%)	26.86	38.93	***
Systolic blood pressure (mm Hg)	127.54 ± 17.43	134.63 ± 20.43	***
Diastolic blood pressure (mm Hg)	75.83 ± 10.69	79.10 ± 10.46	***
Arterial hypertension (%)	22.35	58.72	***
PWV (m/s)	8.80 ± 1.89	10.26 ± 2.13	***
Body mass index (kg/m2)	26.41	27.80	***
Glycaemia (mg/dl)	97.96 ± 24.47	119.94 ± 50.60	***
Diabetes mellitus (%)	3.14	23.15	***
Total cholesterol (mg/dl)	217.72 ± 44.73	205.39 ± 48.14	**
HDL (mg/dl)	57.15 ± 17.04	39.74 ± 9.75	***
LDL (mg/dl)	114.70 ± 37.27	119.02 ± 44.08	NS
Triglycerides (mg/dl)	130.73 ± 85.71	192.97 ± 142.96	***
Dyslipidaemia (%)	11.76	70.81	***

Values presentation: mean ± SD; *P < 0.05, **P < 0.005, ***P < 0.0001, NS not significant P > 0.05; PWV, pulse wave velocity; HDL, high-density lipoprotein; LDL, low-density lipoprotein.

### Genetic and biochemical analyses

Genomic DNA was extracted from an 80 μl aliquot of whole blood using standard phenol/chloroform methodologies with ethanol precipitation. The *ACE *I/D gene alleles (D and I) were identified by PCR amplification as previously described [15]. In order to reduce mistyping of ID heterozygotes as DD homozygotes, a re-amplification was carried out in all identified DD homozygotes using an internal primer specific for the I allele [15]. The other SNPs were identified following previously established PCR-RFLP conditions: *AT1R *A1166C [16], *AGT *M235T [17], *AGT *T174M [18] and *ACE *A11860G [19]. Amplification and digestion results were submitted to electrophoresis in silver stained T9C5 polyacrilamide gels.

### Statistical analysis

In order to analyze the relative influence of genotypes and biochemical parameters among gender, the two groups (controls and patients) were each subdivided into male and female subgroups. Further grouping according to age (45 or less years old) was performed, aiming to analyse the association between genetics and early onset CAD. Individuals aged ≥ 45 years were not included in this sub-analysis because no data about the age of CAD onset was obtained. Basic genetic parameters such as allele and genotype frequencies at each locus, proportion of individual heterozygous samples (direct count heterozygosity as well as the unbiased estimate) and population differentiation were calculated using Genepop v3.1d [20] and Arlequin [21]. Haplotype frequencies were calculated using the PHASE 2.0 software [22]. The overall genetic diversity, within each group diversity and the amount among groups were also calculated. Deviation from Hardy-Weinberg equilibrium per population and locus was calculated according to Weir and Cockerham FIS estimator using FSTAT v.2.9.3., with a Bonferroni correction for all significance levels [23]. Within all groups of subjects, distribution of allele and genotype frequencies and their differences were calculated using χ^2 ^tests. Associated probabilities (P) were calculated applying Fisher's exact test adjusted for multiple comparisons of associated genotypes. To test the significance of association between genotypes at pairs of loci in each sample we used a log-likelihood ratio G-statistic as implemented in Genepop. The relative odds ratio (OR) and 95% confidence interval of relative CAD risk for any of the genetic polymorphisms and biochemical and behaviour markers, was assayed by logistic regressions using the SPSS package. To analyse possible positive or negative interactions between classical risk factors of CAD and genetic polymorphisms we used a 4 × 2 table approach and epiInfo 3.4.3 to calculate ORs, respective 95% confidence intervals and two-tailed p values, as well as synergy measures in additive (SI) and multiplicative models (SIM) [24-26]. It was assumed that unexposed individuals without the susceptibility genotype have a certain background risk for disease (OR_00 _is assumed to be 1); OR_10 _refers to the relative risk for disease among people without the susceptibility genotype for disease but exposed to the environmental risk factor relative to those with neither the susceptibility genotype nor exposure; OR_01 _refers to the relative risk among people with the susceptibility genotype who are not exposed to the risk factor relative to those with neither the susceptibility genotype nor exposure; OR_11 _is the ratio of disease risk among exposed people with susceptibility genotype to diseased risk among unexposed people without the susceptibility genotype. These ORs were then used in the calculation of synergy indexes: SI = (OR_11_-1)/(OR_10_+OR_01_-2), SIM = OR_11_/(OR_10 _× OR_01_) [25,26]; the relative excess risk due to interaction, RERI = OR_11_-OR_10_-OR_01_+1 [26]; and the attributable proportion of the disease due to interaction, AP = RERI/OR_11 _[26].

## Results

### Classical risk factors

As expected, both the control and patient group showed differences in the biochemical markers and other conventional risk factors analysed (Table 1). Systolic and diastolic blood pressure, dyslipidaemia, arterial hypertension, diabetes mellitus, triglycerides and a previous record of CAD in the family are much higher in CAD patients then in controls. In average, LDL was also higher in the CAD group and HDL values were lower in the patients group, as well as total cholesterol. We performed a multivariate logistic regression analysis using the variables included in table 1 and also the putative risk genotypes of RAS polymorphisms. We found HDL (OR = 0.91 95%CI: 0.89–0.93, p < 0.0001), glycaemia (OR = 1.01 95%CI: 1.01–1.02, p < 0.0001), CAD history (OR = 2.14 95%CI: 1.36–3.35, p = 0.001), smoking habit (OR = 1.81 95%CI: 1.11–2.93, p = 0.017), dyslipidaemia (OR = 13.18, 95%CI: 8.32–22.14, p < 0.0001) and *ACE *I/D DD polymorphism (OR = 1.72, 95%CI: 1.08–2.75, p = 0.022) to be independently related to CAD.

### Allele and genotype distribution

Table 2 presents the distribution of genotypes for the 5 loci in controls and patients. Genotypes at both *ACE *loci show statistically different distributions for both the overall analysis and male subgroup. Only the group of patients is in Hardy-Weinberg equilibrium at each locus and overall as the control group is not at HWE at locus *ACE *I/D (χ^2^df_1 _= 7.55, P < 0.01). As expected, mutations at the *AGT *and *ACE *loci show strong significant linkage disequilibrium within each gene (P < 0.0001) for both groups, even after Bonferroni correction. The group of patients also shows significant genotypic disequilibrium at *AGT*235/*AT1R *(P < 0.03). An exact G-test for population differentiation gave an overall value of 0.00238 in which the loci contributing significantly to the differentiation between the two samples are both *ACE *polymorphisms (χ^2 ^test, P < 0.0001 and P < 0.01 for *ACE *I/D and *ACE*11860, respectively). Thus taking in consideration all loci combined, both populations are significantly different concerning the genic differentiation (χ^2 ^df_10 _= 24.082, P = 0.007).

**Table 2:** Distribution of genotypes between patients and controls

Genotype		Whole population (n = 808)			Males (n = 530)			Females (n = 278)			≤ 45 subgroup (n = 275)		
		CAD (n = 298)	Control (n = 510)	P	CAD (n = 235)	Control (n = 295)	P	CAD (n = 63)	Control (n = 215)	P	CAD (n = 64)	Control (n = 111)	P

*AGT*235	MM	86(28.86)	148(29.02)	NS	63(26.81)	87(29.49)	NS	23(36.51)	61(28.37)	NS	19(29.69)	63(29.85)	NS
	MT	155(52.01)	275(53.92)		124(52.77)	155(52.54)		31(49.21)	120(55.81)		31(48.44)	111(52.61)	
	TT	57(19.13)	87(17.06)		48(20.42)	53(17.97)		9(14.28)	34(15.81)		14(21.87)	37(17.54)	
*AGT*174	TT	235(78.86)	400(78.43)	NS	185(78.72)	229(77.63)	NS	50(79.36)	171(79.53)	NS	47(73.44)	160(75.83)	NS
	TM	59(19.80)	107(20.98)		46(19.57)	65(22.03)		13(20.63)	42(19.53)		16(25.00)	48(22.75)	
	MM	4(1.34)	3(0.59)		4(1.70)	1(0.34)		0(0)	2(0.93)		1(1.56)	3(1.42)	
*AT1R*1166	AA	175(58.72)	291(57.06)	NS	137(58.30)	179(60.68)	NS	38(60.32)	112(52.09)	NS	35(54.69)	110(52.13)	NS
	AC	106(35.57)	193(37.84)		84(35.74)	104(35.25)		22(34.92)	89(41.40)		24(37.50)	86(40.76)	
	CC	17(5.70)	26(5.10)		14(5.96)	12(4.07)		3(4.76)	14(6.51)		5(7.81)	15(7.11)	
*ACE *I/D	II	38(12.75)	84(16.47)	**	29(12.34)	47(15.93)	***	9(14.29)	37(17.21)	NS	8(12.50)	33(15.64)	NS
	ID	137(45.97)	282(55.29)		108(45.96)	178(60.34)		29(46.03)	104(48.37)		28(43.75)	119(56.40)	
	DD	123(41.28)	144(28.24)		98(41.70)	70(23.73)		25(39.68)	74(34.42)		28(43.75)	59(27.96)	
*ACE*11860	AA	54(18.12)	113(22.16)	**	41(17.45)	65(22.03)	*	13(20.63)	48(22.33)	NS	11(17.19)	43(20.38)	NS
	AG	135(45.30)	255(50.00)		109(46.38)	155(52.54)		26(41.27)	100(46.51)		28(43.75)	111(52.61)	
	GG	109(36.58)	142(27.84)		85(36.17)	75(25.42)		24(30.10)	67(31.16)		25(39.06)	57(27.01)	

Values presentation: n(relative frequencies%); *P < 0.05, **P < 0.005, ***P < 0.0001, NS not significant P > 0.05.

Male patient and control groups show significant differences in allele content at both *ACE *I/D and *ACE*11860 loci (P < 0.0001 and P < 0.05, respectively). This is also reflected on the distribution of genotypes across *ACE *I/D which is highly significant in a G-like test (P < 0.003).

Analysing possible genotype associations of *ACE *polymorphisms and CAD development we obtained significant results when testing *ACE *I/D DD and *ACE*11860 GG, while *ACE *I/D ID genotype showed to decrease CAD risk (Table 3). The male population analysis resulted even more significant for these polymorphisms and no significant results where found in the female subgroup. *ACE *I/D DD genotype was shown to increase the risk of early onset CAD.

**Table 3:** Allele, genotype and haplotype association between ACE polymorphism and CAD and RAS combined set of alleles association with CAD

	**Whole population**	**Males**	**Females**	**≤ 45 subgroup**
**Allele**				

*ACE*11860 G	1.312(1.07–1.60)**	1.387(1.09–1.77)**	NS	NS
*ACE *I/D D	1.414(1.15–1.74)***	1.562(1.22–2.00)***	NS	NS

**Genotype**				

*ACE*11860 GG	1.560(1.16–2.11)**	1.713(1.18–2.49)**	NS	NS
*ACE *I/D DD	1.795(1.34–2.41)***	2.283(1.57–3.31)***	NS	1.880(1.05–3.36)*

***ACE *****haplotype (*****ACE*****11860/*****ACE*****I/D)**				

A/I	0.670(0.54–0.83)***	0.660(0.51–0.85)**	0.653(0.43–1.00)*	NS
	CAD = 191, C = 425	CAD = 155, C = 251	CAD = 39, C = 175	CAD = 42, C = 179
A/D	1.526(1.03–2.27)*	NS	2.434(1.20–4.94)*	NS
	CAD = 47, C = 55	CAD = 35, C = 34	CAD = 14, C = 21	CAD = 8, C = 18
G/I	NS	NS	8.372 (2.13–32.87)**	NS
	CAD = 20, C = 23	CAD = 12, C = 21	CAD = 7, C = 3	CAD = 3, C = 6
G/D	1.256(1.03–1.53)*	1.440(1.13–1.84)**	NS	NS
	CAD = 335, C = 520	CAD = 269, C = 283	CAD = 66, C = 231	CAD = 75, C = 219

**RAS combined set of alleles**^**1**^**(*****ACE*****11860/*****ACE*****I/D/*****AGT*****174/*****AGT*****235/*****AT1R*****)**				

A/I/T/M/A	0.698(0.53–0.93)*	0.663(0.50–0.88)**	NS	NS
	CAD = 76, C = 179	CAD = 96, C = 164		
A/I/T/T/A	0.727(0.55–0.97)*	NS	NS	NS
	CAD = 76, C = 173			
A/D/M/T/A	NS	3.804(1.02–14.13)*	NS	NS
		CAD = 9, C = 3		
A/I/M/T/C	0.324(0.11–0.95)*	NS	NS	NS
	CAD = 4, C = 20			
G/D/T/T/A	1.459(1.06–2.02)*	1.400(1.01–1.95)*	NS	2.468(1.21–5.04)*
	CAD = 71, C = 88	CAD = 87, C = 82		CAD = 14, C = 20

Values presentation: OR(95% CI); *P < 0.05, **P < 0.005, ***P < 0.0001, NS not significant P > 0.05; CAD, number of CAD patients; C, number of control subjects.

^1^Only combined set of alleles present in more than 10 individuals and yielding significant results are shown.

### Haplotype analysis

Within the *ACE *gene, the haplotype *ACE*11860 A/*ACE *I/D I was found to provide a decreased risk of developing CAD, except for the ≤ 45 subgroup analysis. An association between *ACE*11860 G/*ACE *I/D D and CAD was found in the whole population and male subgroup analysis, while *ACE*11860 G/*ACE *I/D I was associated with the disease in females (Table 3). No association was found when analysing the *AGT *gene haplotypes (results not shown). Considering all genes involved in the RAS system, only five out of nineteen obtained combinations yielded significant associations with CAD (Table 3), especially a male driven decreased risk provided by the *ACE*11860 A/*ACE *I/D I/*AGT*174T/*AGT*235 M/*AT1R *A combination and an increased probability of early onset CAD provided by *ACE*11860 G/*ACE *I/D D/*AGT*174 T/*AGT*235T/*AT1R *A.

### Interaction between RAS polymorphisms and classical risk factors

The analysis of the possible positive/negative association between genotypes and classical risk factors is expressed in Tables 4 and 5, except for polymorphism *ACE*11860 GG, which rendered highly similar results to the *ACE *I/D analysis, so the results are not shown, and *AGT*174, with insufficient polymorphic data to perform this analysis. Regarding *AGT*235 TT or *AT1R *CC, we analysed only the whole population data, because the subdivision in sex or age classes rendered insufficient number of individuals to perform statistical analysis. Regarding the conventional risk of subjects unexposed to both classical risk factor and genetic risk (reference category) as being 1.0, the OR estimating the effect of joint exposure to hypertension and *ACE *I/D DD, *AGT*235 TT or *AT1R *CC was significantly higher than the ORs estimating the effect of each factor in the absence of the other. The synergy index in early onset CAD analysis (*ACE *I/D DD) was above 9, indicating a departure from an additive relation. In this group the proportion of CAD attributable to the interaction of hypertension and *ACE *I/D DD was as high as 85%. The genotypes *AT1R *CC in the overall analysis, or *ACE *I/D DD in all subgroups, interact with smoking habit to develop CAD, showing more than a multiplicative effect in females (SI = 0.86, SIM = 1.77, AP = 0.13). The risk provided by obesity was found to be positively reinforced by *AT1R *CC in the whole population analysis and *ACE *I/D DD in all subgroups, particularly in females (SI = 2.82, SIM = 1.79; AP = 0.46) and ≥ 45 subgroup (SI = 4.13, SIM = 2.36, AP = 0.65). Performing the same analysis regarding diabetic individuals, significant results were found for *ACE *I/D DD carriers in the whole population analysis and a strong enhancement was found in females (SI = 5.04, SIM = 4.11, AP = 0.79), but not in males (SI and SIM < 1, AP < 0). None of the 2 polymorphisms presented in table 5 were found to interact with diabetes; nevertheless we must point that all 6 individuals carrying the combination *AT1R *CC and diabetes were CAD patients. The join presence of dyslipidaemia and *ACE *I/D DD interacts significantly in CAD onset, except in females (SI and SIM < 1, AP = -0.15). This combination was shown to be accountable for 89% of the disease in the = 45 years subgroup (SI = 9.19, SIM = 9.46, AP = 0.89). All 12 individuals with *AT1R *CC genotype and dyslipidaemia were found to be CAD patients, even though no significant results were obtained due to statistical constraint.

**Table 4:** Synergistic effect of ACE I/D DD genotype and classical risk factors in CAD patients and controls

Classical risk factor	*ACE *I/D DD	Whole population			Males			Females			≤ 45 subgroup		
		CAD	Control	OR(95%CI)	CAD	Control	OR(95%CI)	CAD	Control	OR(95%CI)	CAD	Control	OR(95%CI)
		
Hypertension													

0	0	63	256	1	54	143	1	9	113	1	24	132	1
0	1	43	99	1.76(1.10–2.84)*	39	43	2.40(1.36–4.25)**	4	56	0.90(0.22–3.38)NS	11	55	1.10(0.47–2.55)NS
1	0	112	110	4.14(2.78–6.17)***	83	82	2.68(1.69–4.25)***	29	28	13.00(5.16–33.64)***	12	20	3.30(1.32–8.25)*
1	1	80	45	7.22(4.46–11,73)***	59	27	5.79(3.22–10.46)***	21	18	14.65(5.33–41.40)***	17	4	23.38(6.59–90.92)***
		SI = 1.59; SIM = 0.99; RERI = 2.32; AP = 0.32			SI = 1.56; SIM = 0.90; RERI = 1.71; AP = 0.30			SI = 1.15; SIM = 1.25; RERI = 1.75; AP = 0.12			SI = 9.32; SIM = 6.44; RERI = 19.98; AP = 0.85		
Smoking													

0	0	112	269	1	76	153	1	36	116	1	11	106	1
0	1	70	104	1.62(1.09–2.39)*	48	47	2.06(1.23–3.45)*	22	57	1.24(0.64–2.41)NS	9	37	2.34(0.81–6.72)NS
1	0	63	97	1.56(1.04–2.34)*	61	72	1.71(1.07–2.71)*	2	25	0.26(0.04–1.21)NS	25	46	5.24(2.24–12.46)***
1	1	53	40	3.18(1.95–5.21)***	50	23	4.38(2.40–8.02)***	3	17	0.57(0.12–2.23)NS	19	22	8.32(3.21–21.96)***
		SI = 1.85; SIM = 1.26; RERI = 1.00; AP = 0.31			SI = 1.91; SIM = 1.24; RERI = 1.61; AP = 0.37			SI = 0.86; SIM = 1.77; RERI = 0.07; AP = 0.13			SI = 1.31; SIM = 0.68; RERI = 1.74; AP = 0.21		
Obesity													

0	0	126	295	1	99	180	1	27	115	1	30	135	1
0	1	88	122	1.69(1.18–2.42)**	72	59	2.22(1.42–3.46)**	16	63	1.08(0.51–2.27)NS	22	55	1.80(0.91–3.55)NS
1	0	49	71	1.62(1.04–2.51)*	38	45	1.54(0.91–2.60)NS	11	26	1.80(0.73–4.39)NS	6	17	1.59(0.51–4.77)NS
1	1	35	22	3.72(2.03–6.87)***	26	11	4.30(1.93–9.71)***	9	11	3.48(1.18–10.24)*	6	4	6.75(1.56–30.78)*
		SI = 2.07; SIM = 1.36; RERI = 1.41; AP = 0.38			SI = 1.88; SIM = 1.26; RERI = 1.54; AP = 0.36			SI = 2.82; SIM = 1.79; RERI = 1.60; AP = 0.46			SI = 4.13; SIM = 2.36; RERI = 4.36; AP = 0.65		
Diabetes													

0	0	123	342	1	101	208	1	22	134	1	31	151	1
0	1	89	134	1.85(1.30–2.63)**	75	61	2.53(1.64–3.91)***	14	73	1.17(0.53–2.56)NS	25	59	2.06(1.08–3.95)*
1	0	52	24	6.02(3.46–10.55)***	36	17	4.36(2.25–8.54)***	16	7	13.92(4.69–42.75)***	5	1	24.35(2.61–570.81)**
1	1	34	10	9.45(4.33–21.12)***	23	9	5.26(2.22–12.78)***	11	1	67.00(8.19–1458.07)***	3	0	--
		SI = 1.44; SIM = 0.85; RERI = 2.58; AP = 0.27			SI = 0.87; SIM = 0.47; RERI = -0.63; AP = -0.12			SI = 5.04; SIM = 4.11; RERI = 52.91; AP = 0.79			--		
Dyslipidaemia													

0	0	52	327	1	47	197	1	5	130	1	16	144	1
0	1	35	123	1.79(1.08–2.96)*	31	57	2.28(1.28–4.05)**	4	66	1.58(0.34–7.05)NS	6	58	0.93(0.31–2.70)NS
1	0	123	39	19.83(12.16–32.47)***	90	28	13.47(7.68–23.77)***	33	11	78.00(22.86–287.79)***	20	8	22.50(7.80–67.21)***
1	1	88	21	26.35(14.58–48.04)***	67	13	21.60(10.54–45.02)***	21	8	68.25(18.00–282.98)***	22	1	198.00(25.16–4210.95)***
		SI = 1.29; SIM = 0.74; RERI = 5.73; AP = 0.22			SI = 1.50; SIM = 0.70; RERI = 6.85; AP = 0.32			SI = 0.87; SIM = 0.55; RERI = -10.33; AP = -0.15			SI = 9.19; SIM = 9.46; RERI = 175.57; AP = 0.89		

SI, Rothman's synergy index for interaction; RERI, relative excess risk due to interaction; AP, proportion of disease attributable to interaction; *P < 0.05, **P < 0.005, ***P < 0.0001, NS not significant P > 0.05.

**Table 5:** Synergistic effect of AGT235 MM and AT1R CC genotypes and classical risk factors in CAD patients and controls

Classical risk factor	*AGT*235 MM	Whole population^1^			*AT1R CC*	Whole population^1^		
Hypertension		CAD	Controls	OR(95%CI)		CAD	Controls	OR(95%CI)
				
0	0	88	293	1	0	99	334	1
0	1	18	62	0.97(0.52–1.78)NS	1	7	21	1.12(0.42–2.89)NS
1	0	153	130	3.92(2.77–5.55)***	0	182	150	4.09(2.96–5.66)***
1	1	39	25	5.19(2.88–9.41)***	1	10	5	6.75(2.06–23.27)**
		SI = 1.44; SIM = 1.36; RERI = 1.30; AP = 0.25			SI = 1.79; SIM = 1.47; RERI = 2.54; AP = 0.38			
Smoking								
0	0	145	315	1	0	177	356	1
0	1	37	58	1.39(0.86–2.24)NS	1	5	17	0.59(0.19–1.74)NS
1	0	96	108	1.93(1.36–2.75)**	0	104	128	1.63(1.18–2.27)**
1	1	20	29	1.50(0.79–2.85)NS	1	12	9	2.68(1.03–7.05)*
		SI = 0.38; SIM = 0.56; RERI = -0.82; AP = -0.55			SI = 7.64; SIM = 2.79; RERI = 1.46; AP = 0.54			
Obesity								
0	0	172	352	1	0	201	394	1
0	1	42	65	1.32(0.84–2.07)NS	1	13	23	1.11(0.52–2.34)NS
1	0	69	71	1.99(1.34–2.95)**	0	80	90	1.74(1.22–2.50)**
1	1	15	22	1.40(0.67–2.89)NS	1	4	3	2.61(0.49–14.83)NS
		SI = 0.31; SIM = 0.53; RERI = -0.91; AP = -0.65			SI = 1.89; SIM = 1.35; RERI = 0.76; AP = 0.29			
Diabetes								
0	0	171	396	1	0	201	450	1
0	1	41	80	1.19(0.77–1.84)NS	1	11	26	0.95(0.43–2.05)NS
1	0	70	27	6.00(3.63–9.98)***	0	80	34	5.27(3.34–8.33)***
1	1	16	7	5.29(2.00–14.47)***	1	6	0	--
		SI = 0.83; SIM = 0.74; RERI = -0.90; AP = -0.17			--			
Dyslipidaemia								
0	0	73	370	1	0	82	424	1
0	1	14	80	0.89(0.45–1.71)NS	1	5	26	0.99(0.32–2.83)NS
1	0	168	53	16.07(10.59–24.43)***	0	199	60	17.15(11.62–25.37)***
1	1	43	7	31.14(12.81–79.15)***	1	12	0	--
		SI = 2.01; SIM = 2.18; RERI = 15.18; AP = 0.49			--			

SI, Rothman's synergy index for interaction; RERI, relative excess risk due to interaction; AP, proportion of disease attributable to interaction; *P < 0.05, **P < 0.005, ***P < 0.0001; ^1^We performed no further subdivision when analysing these polymorphisms because several classes resulted statistically impossible to analyse due to the reduced number of individuals there included.

## Discussion

Previous studies have focused on the association of any of the *ACE*, *AT1R *and *AGT *gene polymorphisms with coronary events of several degrees, related or not with MI and essential hypertension. Up to know there is no consistent genetic pattern that may link a given haplotype to the risk of developing a CAD related syndrome or at least to make an individual more prone to be affected. All 5 gene polymorphisms surveyed here promote phenotypic variants on known mechanisms leading to changes in the biochemical status of an individual. For example, the *ACE *I/D variant is linked to ACE activity [10] and *AGT *M235T to different angiotensinogen plasma levels [3,14].

By evaluating the distribution of RAS gene polymorphisms in a series of patients undergoing coronary angiography, the present study has shown that only mutations at *ACE *gene seem to be linked to CAD and even these are apparently male-linked because no such association was visible in females. The association of *ACE *I/D DD genotype with CAD in men but not in women, has been reported previously in several studies [27,28]. While our patient subpopulation was in Hardy-Weinberg equilibrium for I/D polymorphism of *ACE *gene, the control subpopulation was not, due to an excess of heterozygotes ID. Similar results were previously reported [5,29] and may be interpreted as a case of heterosis, or even confirm the higher risk homozygotes DD have to develop CAD.

The deletion polymorphism of the *ACE *gene has been shown to be associated with both CAD and MI [5,30,31]. The mechanism by which the *ACE *I/D or *ACE*11860 genotypes may predispose an individual to the development of MI remains unclear. *ACE *is responsible for the conversion of angiotensin I to the peptide precursor angiotensin II, which has been implicated in the pathogenesis of atherosclerosis [32,33]. In contrast, other studies concluded that *ACE *polymorphisms did not influence the development of MI or other manifestations of CAD [34,35]. There are several possible reasons for these discrepancies: besides the different genetic backgrounds of the study populations, some of the studies cited have only minor statistical power or the associations between *ACE *gene polymorphisms and CAD or MI have been restricted to relatively small subgroups. Even more, in some studies, the presence or absence of CAD was not determined by angiography and might even have used false-negative control populations. These findings also stress the necessity of considering ethnic factors in the assessment of genetic risk identifiers.

The total lack of association between the 2 *AGT*s and *AT1R *polymorphisms and CAD is in agreement with other studies [14,36] that found no relation between these polymorphisms with CAD albeit a strong association between *AGT *variants and angiotensinogen levels. Nevertheless, our study revealed a significant influence of the A allele and AA genotype of *AT1R *upon an increase in carotid-femoral PWV values, which may be regarded as a risk factor for CAD (results not shown).

The presence of the *ACE *I/D I and *ACE*11860 A alleles in *ACE *haplotypes corresponds to the lowest risk of developing CAD in the whole population, male and female subgroups, expressed by ORs lower than 1. When analysing the influence of RAS haplotypes in the development of CAD, it is clear that the major influence comes from both *ACE *polymorphisms. Yet again, the simultaneous presence of *ACE *I/D I and *ACE*11860 A alleles, corresponds to a significant trend towards a decrease in CAD, both in male subgroup and overall analysis. The combined set of RAS alleles *ACE*11860 G/*ACE *I/D D/*AGT*174 T/*AGT*235 T/*AT1R *A was the only one found to significantly increase CAD risk in the whole population analysis. No significant association was found in females, showing once again the interest of separating this type of data between sexes.

Premature CAD is known to have a particularly strong genetic component. Previous data have suggested that genetic factors are more likely to affect young rather than old people [37]. We conducted a population subdivision, analysing separately individuals who developed CAD before the age of 45. We found no significant allele or genotype association between any of the 5 polymorphisms and early onset CAD. The only significant results were found when associating the RAS combined set of alleles with the disease, where individuals under 45 have a 2.47 relative risk of CAD occurrence when in the join presence of *ACE*11860 G/*ACE *I/D D/*AGT*174 T/*AGT*235 T/*AT1R *A. Here we must point the impossibility to further subdivide this group according to sex, due to small sample size, as a limitation to perform a more thorough analysis. One should be aware that in almost all case-control studies, particularly those involving haplotype analysis, problems related to multiple comparisons (even when statistically corrected), the potential influence of genetic and environmental factors not considered, limited sample size for subgroup analysis and the possible inclusion of patients with silent CAD in the control group should be taken into account. The fact that the control and CAD patients groups are not sex and age matched may also be regarded as a constraint in the study design.

The complex aetiology around cardiovascular disorders and the multiple environmental conditionings are most of the times not evaluated together. In most cases, CAD has a multifactorial genetic basis, involving a number of genes and environmental factors interacting to determine whether or not the disease will develop. Therefore, the inherited genes generally predispose to a greater or lesser extent of CAD, but it is the environmental factors (e.g. cigarette smoking, obesity, hypertension, sedentarism) interacting with the individual's genotype that determine whether or not CAD will develop. We performed further analysis in order to determine whether the simultaneous presence of genetic polymorphisms and well established risk factors – hypertension, smoking habit, obesity, diabetes and dyslipidaemia – would enhance the effect in CAD onset of these last. Some interesting results were obtained, particularly in the subgroups analysis. As both *ACE *polymorphisms have shown to be in strong linkage disequilibrium, we chose not to show the results of this analysis for *ACE*11860 GG. They are very alike those presented in table 4 as the exact same associations were found with similar, yet slightly lower values. We found a proportion of CAD attributable to the interaction between hypertension and *ACE *I/D DD genotype around 30% for the whole population and male subgroup analysis. In the early onset CAD analysis, this proportion goes up to 85%. The vascular risk factors that might be related to serum ACE activity are not yet fully understood. Nevertheless, a previous study has shown a correlation between male sex and history of hypertension with serum ACE activity [38], which may partially support our findings. This effect was also found when considering *AGT*235 TT or *AT1R *CC as the risk genotypes (SI = 1.44, SIM = 1.36, AP = 0.25; SI = 1.79, SIM = 1.47, AP = 0.38, respectively). Smoking may increase ACE levels by means of a nicotine enhancement of *ACE *gene expression [39]. A large population based study [40] found a positive association between the D allele of the I/D polymorphism and carotid artery thickness among smokers: individuals carrying only one of the risk factors did not show significant differences in artery thickness when compared to non-smokers with II genotype, while carriers of both risk factors had significantly higher artery thickness. In our study we found a synergistic effect due to smoking and *ACE *I/D DD join presence, which is in accordance with a previous study [41]. Moreover, these authors found a positive association between total high cholesterol, high LDL or overweight/obesity and the DD genotype. In the present report we also found this association, concerning dyslipidaemia and obesity. This synergy effect is particularly striking when considering the early onset CAD subgroup analysis (obesity: SI = 4.13, SIM = 2.36, AP = 0.65; dyslipidaemia: SI = 9.19, SIM = 9.46, AP = 0.89). Obesity and hypercholesterolemia have been shown to play a role in *ACE *gene expression, but also in other RAS intervenients, such as *AGT *or *AT1R *[42]. Nevertheless, we found no association between obesity and *AGT*235 TT or dyslipidaemia and *AT1R *CC, even though all 12 dyslipidaemic individuals carrying *AT1R *CC were CAD patients. A similar study, analysing *AGT*235 T allele synergistic effect with several risk factors, found a positive association with hypercholesterolemia but neither with hypertension nor smoking [43]. The association between *ACE *I/D polymorphism and Type 2 diabetes has been rather controversial. Some studies have affirmed a clear association between DD genotype and the disease in Caucasians [44] while others have excluded this hypothesis [45]. We found a female driven synergistic effect of DD genotype together with diabetes (SI = 5.04, SIM = 4.11, AP = 0.79).

## Conclusion

The most relevant findings of this research are based on the influence of RAS gene polymorphisms evaluated together with the presence of the main classical risk factors for CAD, showing how at least two polymorphisms – *ACE *I/D and *ACE*11860 A/G – interact synergistically with them in CAD onset. Taking into account that CAD is a multifactorial disorder driven by numerous environmental, behavioural and genetic components interacting together, our results seem to corroborate the hypothesis that RAS gene polymorphisms may indeed enhance the influence of traditional risk factors in CAD development. The sample used in this study is also a novelty because it is based on the population of Madeira Island that until the beginning of the 20th century was a relatively isolated island. To our knowledge this analysis on such a population had not yet been performed. Madeiran inhabitants with a history of a small founder population, long lasting isolation and population bottleneck represent an exceptional resource in the identification of genes involved in the pathogenesis of multifactorial diseases.

## Competing interests

The authors declare that they have no competing interests.

## Authors' contributions

AIF performed this work as part of her PhD thesis, under guidance of AB and AC. AB, IM and RPR conceived the idea for the study. Lab and clinical data were performed under AB, IM and AC guidance. All authors performed data analysis, interpretation and discussion of results and also read and approved the final manuscript.

## Pre-publication history

The pre-publication history for this paper can be accessed here:


